# Simulation-based Training Changes Attitudes of Emergency Physicians Toward Transesophageal Echocardiography

**DOI:** 10.5811/westjem.20788

**Published:** 2025-03-15

**Authors:** Michael Danta, Alyssa Y. Nguyen-Phuoc, Suman Gupta, Aneri Sakhpara, Jeanette Kurbedin, Errel Khordipour, Antonios Likourezos, Lawrence Haines, Amish Aghera, Jefferson Drapkin, Judy Lin

**Affiliations:** * Maimonides Medical Center, Department of Emergency Medicine, Brooklyn, New York; † University of California San Francisco-Fresno, Department of Emergency Medicine, Fresno, California; ‡ Regional Medical Center of San Jose, San Jose, California; § Baylor Scott & White All Saints, Department of Emergency Medicine, Fort Worth, Texas

## Abstract

**Objective:**

The American College of Emergency Physicians recommends that transesophageal echocardiography (TEE) be used to “maintain the standard of ultrasound-informed resuscitation” in cardiac arrest. To date, no standards exist on how to train emergency physicians (EP) on TEE use in the emergency department (ED). We propose a novel educational paradigm using simulation to train EPs on the use of TEE in cardiac arrest.

**Methods:**

A total of 63 EPs at a single-center academic teaching hospital participated in a 90-minute simulation-based education session to summarize the use of TEE in cardiac resuscitation and practice related procedural skills. The session consisted of a simulated cardiac arrest scenario using both transthoracic echocardiography (TTE) and TEE and hands-on practice on a high-fidelity TEE task trainer. Participants filled out anonymous surveys before and after the training session, which evaluated their subjective attitudes toward TEE, knowledge of its role in cardiac arrest, and perceived efficacy of the curriculum in introducing the modality.

**Results:**

Survey results indicated fewer perceived barriers to performing TEE in resuscitation after completion of the course, with statistically significant decreases in the following: not understanding image acquisition (85.5% pre vs 27.4% post; *P*<0.001), interpretation (66.1% pre vs 25.8% post; *P*<0.001), indications (29.0% pre vs 0.0% post; *P*<0.001), contraindications (35.5% pre vs. 3.2% post; *P*<0.001), and the potential benefit for the patient (24.2% pre vs 3.2% post; *P* <0.001). Finally, 68% of EPs stated they were “extremely likely” to use TEE in cardiac arrest with the availability of assistance from a credentialed attending.

**Conclusion:**

The survey responses suggest that a short, simulation-based course can generate interest in the incorporation of TEE in cardiac resuscitation as well as overcome many of the perceived barriers regarding TEE. Moreover, they suggest that the participating academic EPs would be interested in using TEE in critical patients in the future when available.

## BACKGROUND

The American College of Emergency Physicians (ACEP) recommended in 2017 that transesophageal echocardiography (TEE) be used to “maintain the standard of ultrasound-informed resuscitation in the scenario of cardiac arrest.”^1^ Whereas traditionally transthoracic echocardiography (TTE) has been employed by emergency physicians (EP) in resuscitation scenarios, its utility is limited by potentially poor image quality due to suboptimal windows. Additionally, studies have shown that the use of TTE during cardiac arrest increases pulse check duration.^2^,^3^ In comparison, TEE has been demonstrated to decrease compression pauses and improve visualization of cardiac structures for reversible causes of cardiac arrest.^4^ Studies in animal models also suggest that TEE can be used to guide cardiac compressions for maximal cardiac output, potentially improving mortality outcomes.^5^ Current evidence shows TEE is a safe procedure with the risk of perforation limited to 0.01% and an overall mortality rate of 0.0098%.^6^,^7^

Despite TEE being identified as potentially useful during resuscitation, there are many logistical challenges to establishing TEE at an institution, including equipment availability, sterilization protocols, training, and credentialing. Even within departments with established TEE programs, however, its use varies widely. This may be due to a lack of evidence-based standards on how to train EPs on TEE use, as well as other perceived barriers that have not yet been identified. Prior studies on TEE education have involved small groups of learners, primarily trainees; moreover, they have focused on learners with prior advanced ultrasound training.^8^ Furthermore, the understanding of TEE in the larger emergency medicine (EM) community is limited, with no current studies identifying perceptions and gaps in knowledge of TEE in the general EP population. Adoption of TEE by emergency departments (ED) has been hindered by a multitude of factors, many of which may not be fully recognized.

Compared to traditional educational modalities, the use of simulation allows learners to expose underlying practice patterns and decision-making frameworks, while also establishing a formal mechanism to de-bias error-prone behaviors via structured debriefing.^9^ This allows participants to conceptualize new ideas to be implemented in practice.^10^ Currently, there is little literature on how to incorporate simulation into TEE education for practicing and non-ultrasound-trained EPs. Our objective in this study was to employ a simulation-based education course to train a large and diverse group of EPs at an academic ED, including non-ultrasound fellowship-trained EPs, to study the prevailing barriers to adoption of TEE and the effect of simulation to bridge those barriers.

## METHODS

### Study Design, Setting, and Participants

This was a prospective, single-center, cross-sectional study conducted in a tertiary-care teaching hospital with an EM residency, clinical simulation fellowship, and emergency ultrasound fellowship. We created a simulation-based course to introduce attending EPs to the use of TEE in cardiac resuscitation. Study subjects were a convenience sample of all employed attending EPs in the institution. Subjects participated in a 90-minute course consisting of a simulation case, debriefing, and hands-on practice using a TEE simulator. Excluded were faculty of the emergency ultrasound and clinical simulation divisions with knowledge of the study objectives. Pre- and post-course surveys were used to evaluate changes in physician attitudes toward TEE. The local institutional review board classified this study as “exempt.”

## COURSE DESIGN

The course was implemented January 2019–July 2020 with groups of two to three learners per session. Sessions were facilitated by at least one ultrasound fellowship-trained faculty and one simulation fellowship-trained faculty with experience in TEE and clinical debriefing, respectively. Prior to the training module, all participants completed one hour of asynchronous TEE education consisting of either video podcasts or continuing medical education from prior conferences.

The course began with a simulated cardiac arrest scenario using the high-fidelity simulator CAE Apollo (Global Technologies, Ltd, Parsippany, NJ). The scenario was designed to reproduce the limitations and pitfalls of TTE in cardiac arrest (eg, poor views and prolonged interruptions in compressions) and highlight several advantages of TEE in guiding resuscitation (eg, continuous compressions with a specific prompt to change hand placement to avoid obstructing the aortic outflow tract and demonstration of organized cardiac activity unseen on TTE). Debriefing focused on linking the participants’ simulated challenges using TTE to similar real-world patient experiences, while specifically documenting the duration of prolonged pauses in compressions during the scenario. The framework was explicitly structured to promote critical reflection with regard to methods of decreasing pulse check duration (eg, Cardiac Arrest Sonographic Assessment protocol^11^) and introduce TEE as a safe, approachable tool to improve practice.

The final phase of the course was to provide individualized hands-on instruction with image acquisition and interpretation on a high-fidelity task trainer Ultrasound Mentor (Surgical Science Sweden AB, Göteborg, Sweden). This skills session was led by an ultrasound fellowship-trained EP credentialed to perform TEE based on ACEP standards for competency. Participants engaged in deliberate practice guided by a procedural checklist to obtain and interpret mid-esophageal four-chamber, mid-esophageal long-axis, bicaval, and transgastric mid-papillary short-axis views.^10^ Clinical contexts incorporated reversible causes such as cardiac tamponade, pulmonary embolism, aortic dissection, and fine ventricular fibrillation.

### Variables

Participants filled out anonymous surveys immediately before and immediately after the training session, evaluating their subjective attitudes toward TEE, knowledge of its potential role in cardiac arrest, and perceived efficacy of the curriculum in introducing the modality.

## DATA ANALYSIS

Surveys were de-identified, kept confidential, and reviewed only by members of the research team. We summarized the data using descriptive statistics (percentages for categorical variables and means with standard deviations for continuous variables). Since we did not record participant identifiers, our analysis assumed independence; a chi-square test was performed to compare pre- and post-session knowledge questions. Levels of significance were tested at *P*<0.05. Analyses were conducted using SPSS v27.0 (SPSS Statistics, IBM Corp, Armonk, NY).

## RESULTS

A total of 63 EPs participated in the TEE simulation course (Table 1), and 62 participants completed both pre- and post-course surveys. The strongest perceived barrier to using TEE before the module was image acquisition. There was a significant decrease in this and all other perceived barriers (image interpretation, indications, contraindications, potential benefits, potential risks) to performing TEE in resuscitation after simulation (Table 2). Participants also evaluated course effectiveness using a modified Likert scale ranging from 1 (“not at all effective”) to 5 (“extremely effective).” Based on this data, participants felt the course was effective in teaching potential benefits of TEE in cardiac arrest with a mean modified Likert score of 4.73. Additionally, 68% of EPs stated they were “extremely likely” to use TEE in cardiac arrest with the availability of assistance from a credentialed attending following participation in the education.

## DISCUSSION

Credentialing and training in TEE have been pursued primarily by ultrasound fellowship-trained faculty.^8^ This study is unique in that it focused on introducing TEE to EPs with a broad range of ultrasound and clinical experience, rather than on training the smaller, specialized subset of ultrasound fellowship-trained physicians. A majority of our subjects (84%) did not have ultrasound fellowship training. Additionally, our study included subjects with a diverse mix of clinical experience: 46% of participants had more than five years of clinical experience. This mix of subjects suggests that even experienced clinicians with established practice patterns responded well to the simulation-based module and were interested in expanding their practice to include TEE.

Using simulation to promote active learning among our cohort allowed for a learner-centered discussion of the advantages and disadvantages of TEE, leading to natural evolution toward positive attitudes regarding the modality. Responses from participants suggest that this simulation-based module effectively eroded potential educational barriers to adopting TEE in the ED. The barrier of not understanding indications of TEE was abolished. Barriers such as technical skill in image acquisition and interpretation were allayed significantly.

Our study faces the inherent limitations of a prospective observational study: Survey results focused on the attitudes and reactions of learners following the module and did not assess participant knowledge or skill. Furthermore, all participants came from a single academic ED with well-established ultrasound and simulation divisions, limiting generalizability to the emergency medicine community at large. It also did not evaluate whether TEE is superior to TTE or compare simulation-based TEE education to other modes of education, such as lecture-based formats or asynchronous learning. However, to evaluate these outcomes it is first important to gain buy-in to the idea of TEE, which we focused on in this study.

Lastly, due to the pre- and post- nature of the study, it is difficult to determine whether this theoretical knowledge would translate to long-term changes in practice. Further directions include working toward ongoing training pathways supported by skills assessment on simulated and live patients. Other future directions include establishing a Standardized Direct Observation Assessment Tool to measure retention and competency of TEE skills over time.

## CONCLUSION

Use of transesophageal echocardiography in cardiac arrest is rapidly evolving and a subject of focus in current ultrasound and critical care literature. It is suggested that TEE may revolutionize the way emergency physicians run cardiac resuscitation by better identifying reversible causes of cardiac arrest, allowing for continuous cardiac monitoring, and optimizing high-quality compressions. Despite these potential benefits, adoption of this cutting-edge modality in the ED has been met with hesitancy, especially among practicing EPs without ultrasound fellowship training. Gaining a thorough understanding of potential educational barriers to the adoption and use of TEE is an important step in the process of implementing a TEE curriculum. This study highlighted prevalent gaps in knowledge and revealed the preconceived barriers to use of TEE in the emergency department through the use of simulation-based training.

## Figures and Tables

**Table 1:** Participant demographics.

Characteristic	Number (%) (N=63)
Sex	
Male	32 (51)
Female	31 (49)
Performed TEE before	
Yes	14 (22)
No	49 (78)
Attending experience	
New (<5 years)	34 (54)
Mid (5–10 years)	9 (14)
Experienced (>10 years)	20 (32)
Ultrasound experience	
Ultrasound trained	5 (8)
Current ultrasound fellow	5 (8)
No ultrasound fellowship training	53 (84)

*TEE*, transesophageal echocardiography.

**Table 2:** Perceived barriers to use of transesophageal echocardiography.

Barrier, n (%)	Pre-course 62 (100)	Post-course 62 (100)	P-value
Do not know how to acquire images	53 (85)	17 (27)	<0.001
Do not know how to interpret images	41 (66)	16 (26)	<0.001
Do not know indications	18 (29)	0 (0)	<0.001
Do not know contraindications	22 (35)	2 (3)	<0.001
Do not know potential benefit	15 (24)	2 (3)	<0.001
Causing patient harm or liability	15 (24)	6 (10)	<0.05
